# The Branching Process: A General Conceptual Framework for Addressing Current Ecological and Evolutionary Questions

**DOI:** 10.3390/life15121910

**Published:** 2025-12-13

**Authors:** Xuhua Xia

**Affiliations:** 1 Department of Biology, University of Ottawa, Ottawa, ON K1N 9A7, Canada; xxia@uottawa.ca; Tel.: +1-613-204-2347; 2 Ottawa Institute of Systems Biology, University of Ottawa, Ottawa, ON K1H 8M5, Canada

**Keywords:** Galton-Watson process, offspring distribution, extinction probability, extinction time, fixation time, random process, evolution, demography

## Abstract

Classical branching-process theory, developed by Galton and Watson in the nineteenth century and later refined by Fisher and Haldane, provides the formal framework for quantifying the fate of new mutants, new viral and bacterial pathogens, new colonization of invasive species, etc. It is a powerful tool to quantify and predict the effect of differential reproductive success on the speciation potential of evolutionary lineages. Here, I revisit the conceptual framework of the branching process, detail its mathematical development over time, tie up a few historical loose strings, illustrate the calculation of the exact extinction probability for the Poisson-distributed reproductive success with the Lambert function (which is often missing in the ecological and evolutionary literature), and highlight the potential applications of the branching process in modern ecology and evolutionary biology, especially in deriving the extinction probability and extinction time. I also highlight a few misconceptions about human demography in the US that can be readily dismissed by applying probability tools such as branching processes.

## 1. Introduction

**Figure 1:**
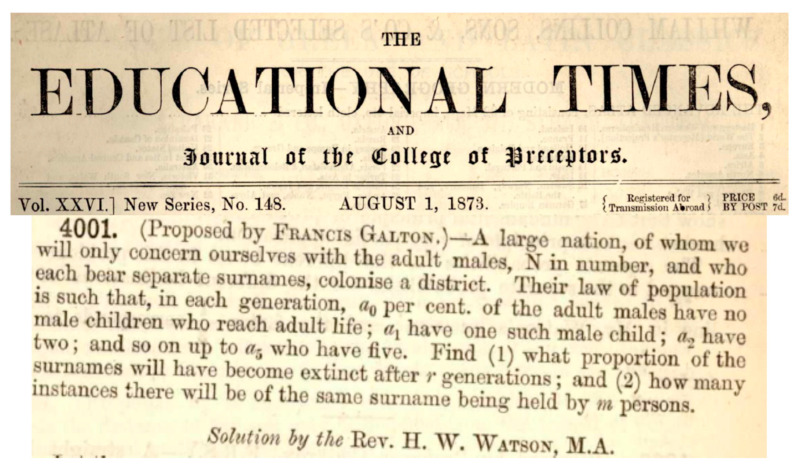
Francis Galton’s question and Henry Watson’s solution.

The branching process is not only “the beautiful theory” in the field of probability [1], but also a key method in evolutionary studies since its formulation [2] (Figure 1). Galton asked two questions. Suppose we start with *N* adult males, each with a distinct surname. In each generation, each man produces *k* adult sons with the probability *a_k_*, with $\sum a_{k}=1$. All adults produce sons independently. What is the chance of a surname whose bearers will perish after *t* generations? The second question is trickier. Galton wanted to have a distribution; that is, after *t* generations, how many surnames will have *m* (=0, 1, 2, …) bearers?

The branching process, which was used by Watson to provide a partial solution to Galton’s questions, has since found applications in ecology and evolution, through the efforts of Fisher [3], Haldane [4], and many others. The basic input to the branching process is a probability mass function specifying the reproductive potential of individuals, and the output consists of extinction probability *Q*, extinction time *T*, and the variance of *T* [5,6]. This paper focuses on the details between the input and the output and highlights its relevance to current research problems in ecology and evolution.

The classical Galton–Watson branching process is a particular case of Markov processes. It can be viewed as a Markov chain with an infinite number of discrete states, which are the possible population sizes. For such a Markov chain, an entry in the transition probability matrix, *P*(*i*,*j*), is the probability for the change from population size *i* to population size *j*. However, there is no upper limit placed on population size. Because the transition probability matrix associated with this Markov chain is infinite-dimensional, the branching process is rarely handled as a Markov chain through a transition probability matrix. Instead, branching processes are studied with probability generating functions.

## 2. A Branching Process with Empirical Reproductive Success

Suppose we follow the queen and the number of her daughter queens of an invasive giant Asian hornet, *Vespa mandarinia*, that almost succeeded in invading North America [7], or the yellow-legged Asian hornet, *V. velutina*, that successfully invaded France and other European countries [8]; this example is also relevant to the release of animals to rescue a declining population [9] or to create a game population for hunting. Will the individuals in the new environment go extinct? How long will it take to go extinct?

I will start with a fictitious data set. Of 100 empirically monitored hornet queens, 50 did not produce any reproducing daughter queens, 30 produced one reproducing daughter queen, and 20 produced two reproducing daughter queens. Thus, a queen produces 0, 1, and 2 reproducing daughter queens with the corresponding empirical probabilities 0.5, 0.3, and 0.2, respectively. The probability mass function (PMF), which is often called the reproduction law or offspring distribution in branching processes, is then:

(1) $$\begin{array}{c} f\left(x=0\right)=0.5\\ f\left(x=1\right)=0.3\\ f\left(x=2\right)=0.2 \end{array}$$

We assume that this PMF stays constant with time in a branching process and applies to every replicating queen. However, one could have branching processes to accommodate queens with different reproducing potentials.

A branching process and its analysis are anchored on a probability generating function (PGF) derived from the PMF. PGF is defined as $G_{X}(s)=\sum_{k=0}^{Max\;k}f\left(k\right)s^{k}$, where *k* is the value that *X* can take, and the subscript *X* can often be omitted without confusion. Given the distribution specified in Equation (1), the PGF is therefore

(2) $$G(s)=0.5s^{0}+0.3s^{1}+0.2s^{2}=0.5+0.3s+0.2s^{2}$$

Note that $G_{X}\left(1\right)=1$; otherwise, the PMF is not valid. The mean and variance of *X* based on the PGF are

(3) $${E\left(X\right)=G\prime }_{X}\left(1\right)=\mu$$

(4) $${Var\left(X\right)=G\prime \prime }_{X}\left(1\right)+G_{X}^{\prime }\left(1\right)-{\left[G_{X}^{\prime }\left(1\right)\right]}^{2}$$

For the PGF in Equation (2), we have

(5) $${G\prime }_{X}(s)=0.3+0.4s;G_{X}^{\prime \prime }(s)=0.4$$

(6) $$E(X)=G_{X}^{\prime }\left(1\right)=0.3+0.4=0.7$$

(7) $${Var\left(X\right)=G\prime \prime }_{X}\left(1\right)+G_{X}^{\prime }\left(1\right)-{\left[G_{X}^{\prime }\left(1\right)\right]}^{2}=0.4+0.7-0.7^{2}=0.61$$

I should add that the empirically estimated *E*(*X*) for the invasive Asian hornet, *V. velutina*, is much greater than 0.7 in this numeric example [8]. In France, where there is only one highly diverged competitor, *V. crabro*, the mean is 3.9. In South Korea, which has already been populated with seven closely related subspecies, the mean is 1.3. This is analogous to metastatic adrenocortical carcinoma cells colonizing different tissues, with a faster growth rate in the liver than in the lungs or lymph nodes [10]. One can use the branching process to model the fate of a hornet queen colonizing a new region or the fate of a cancer cell invading a new tissue.

### 2.1. Extinction Probability Q

What is the probability that an individual who produces offspring according to Equation (1) will leave no descendants in the future? The extinction probability *Q* was derived [2] as the smallest non-negative solution to the following fixed-point equation:

(8) Q=G(Q)

From Equation (2), we have

(9) $$Q=G\left(Q\right)=0.5+0.3Q^{1}+0.2Q^{2}$$

(10) $$0.2Q^{2}-0.7Q+0.5=0$$

This quadratic equation has two roots, $r_{1}=2.5,\;r_{2}=1$. Therefore, *Q* = 1 (which is the smallest non-negative root). This means that any population started from an individual who reproduces according to Equation (1) will certainly go extinct. Fisher [3] recognized the dependence of *Q* on μ. If μ≤1, the mutant will ultimately die out. If μ>1, then there is a non-zero chance of survival. Such findings led to the subsequent classification of subcritical (μ<1), critical (μ=1) and supercritical (μ>1) conditions [5,6]. Under subcritical and critical conditions, Q=1. Under supercritical conditions, Q < 1.

The calculation above allows us to conclude that, if the invasive hornet queens were to produce 0, 1, and 2 daughter queens with probability of 0.5, 0.3 and 0.2, respectively, as specified in Equation (1), then the mean number of daughter queens per queen mother would be only 0.7, and the population would certainly go extinct, even if some founders might be lucky enough to produce multiple daughter queens. However, how long will it take for the invasive hornet to go extinct?

### 2.2. Extinction Time T and Var(T)

A more difficult problem than the extinction probability is the extinction time. The individual (and its descendants) could go extinct at time *t*_1_, *t*_2_, *t*_3_, etc., with their corresponding probabilities, so this extinction time should be characterized as a distribution.

Designate population size at time *t* as *Z_t_*, and the extinction probability of the population by generation *t* as $q_{t}=\mathrm{Pr}(Z_{t}=0)$. We start the process with a single individual at time 0, so $Z_{0}=1$. We can compute $q_{t}$ by iteration using the PGF in Equation (9):

(11) $$q_{t+1}=G(q_{t})$$

For the first few generations:

(12) $$q_{1}=G(q_{0})=G(0)=0.5$$

(13) $$q_{2}=G(q_{1})=G(0.5)=0.5+0.3\times 0.5+0.2\times 0.5^{2}=0.7$$

(14) $$q_{3}=G(q_{2})=G(0.7)=0.808$$

(15) $$q_{4}=G(q_{3})\approx 0.876$$

The functions above introduce us to a mathematical term called composition; e.g., $q_{4}$ is a composition of the function G to itself 4 times. A more descriptive term is iterate [5]; i.e., $q_{4}$ is an iterate of $q_{3}$ or the 4th iterate of $q_{0}$. As *t* increases, $q_{t}$ will increase monotonously towards *Q*. Under subcritical conditions, *Q* = 1, so $q_{t}$ will increase monotonously towards 1.

What is the probability that extinction happens exactly at time *t*; i.e., $\mathrm{Pr}\left(T=t\right)$? This $\mathrm{Pr}\left(T=t\right)$ is simply $q_{t}-q_{t-1}$. Thus

(16) $$\mathrm{Pr}(T=1)=q_{1}-q_{0}=0.5-0=0.5$$

(17) $$\mathrm{Pr}(T=2)=q_{2}-q_{1}=0.7-0.5=0.2$$

(18) $$\mathrm{Pr}(T=3)=q_{3}-q_{2}=0.808-0.7=0.108$$

(19) $$\mathrm{Pr}(T=4)=q_{4}-q_{3}=0.876-0.808=0.068$$

We can calculate *T* by the following equation:

(20) $$T=\sum_{t=1}^{\infty }t\mathrm{Pr}(T=t)=1\times 0.5+2\times 0.2+\cdots =2.3954$$

This equation works because $\sum_{T=1}^{\infty }\mathrm{Pr}(T)=1$. If the extinction probability Q<1, then we should use the weighted average:

(21) $$T=\frac{\sum_{t=1}^{\infty }t\;\mathrm{Pr}\left(T=t\right)}{Q}=\frac{\sum_{t=1}^{\infty }[t\;\left(q_{t}-q_{t-1})\right]}{\sum_{t=1}^{\infty }\left(q_{t}-q_{t-1}\right)}$$

An alternative way of calculating *T* under subcritical conditions is

(22) $$T=\sum_{t=1}^{\infty }\left(1-q_{t}\right)=\left(1-0\right)+\left(1-0.5\right)+\left(1-0.7\right)+\cdots =2.3954$$

The variance of *T* can be computed by the general equation:

(23) $$Var(T)=E(T^{2})-{\left[E(T)\right]}^{2}$$

We already know E(T)=2.3954, so ${\left[E(T)\right]}^{2}=5.7379$. The first term can be calculated in either of the two ways below.

(24) $$E(T^{2})=\frac{\sum_{t=1}^{\infty }t^{2}\mathrm{Pr}\left(T=t\right)}{\sum_{t=1}^{\infty }\mathrm{Pr}\left(T=t\right)}=\sum_{t=1}^{\infty }t^{2}\mathrm{Pr}(T=t)=1^{2}\cdot 0.5+2^{2}\cdot 0.2+\cdots \approx 10.7818$$

(25) $$E(T^{2})=\sum_{t=0}^{\infty }\left(2t+1\right)(1-q_{t})\approx 10.7818(under\;subcriticla\;condition)$$

(26) $$Var(T)=E(T^{2})-{\left[E(T)\right]}^{2}=10.7818-5.7378=5.0439$$

Equations (21) and (24) are generally applicable; i.e., in subcritical, critical, and supercritical conditions. In contrast, Equations (22) and (25) can be used only under subcritical conditions.

This calculation of *T* and its variance allows us to have more informative predictions on the fate of the invasive hornet. Not only will the invasive hornet with the specified reproduction law go extinct, but it also has a probability ofuni 0.95 of going extinct in $2.3954+1.96\times \sqrt{5.0439}$ generations. For the Asian hornets, the number of generations is equivalent to the number of years.

The calculation above is summarized in Table 1, where *t* is the generation, $q_{t}$ in the second column is the probability of extinction by generation *t*, $q_{t+1}-q_{t}$ in the third column is the probability of extinction that occurs exactly in generation *t*; i.e., Pr(T=t). Note that ($q_{t}-q_{t-1}$) is the generation-specific extinction probability, whereas $q_{t}$ is the cumulative probability of extinction. *T* can be calculated either as the weighted average of *t* (weighted by Pr(T=t), i.e., the third column in Table 1) as shown in Equation (20), or as the summation of $1-q_{t}$ in the fourth column in Table 1, as shown in Equation (22). The last column is for calculating $E(T^{2})$ needed for computing *Var*(*T*).

What would be the extinction time *T* and its *Var*(*T*) under supercritical conditions? *T* can be calculated the same way by using Equation (21); i.e., it is conditional on the extinction probability. *Var*(*T*) can also be calculated as before:

(27) $$Var(T)=E(T^{2})-{\left[E(T)\right]}^{2}=\frac{\sum_{t=1}^{\infty }t^{2}\mathrm{Pr}(T=t)}{\sum_{t=1}^{\infty }\mathrm{Pr}(T=t)}-T^{2}$$

For example, in the supercritical case above with E(X)=1.1, we have T≈5.229, Var(T)=43.826. Note that *T* under the supercritical condition could be small when E(X) is large. This is because such a population will either have bad luck and go extinct quickly (i.e., a small *T*) or increase in population size and never go extinct. For example, if the probabilities of producing 0, 1, or 2 offspring per generation are 0.1, 0.4, and 0.5, respectively, then E(X)=1.5. The resulting T=2.159, Var(T)=3.026. This means that the population will either go extinct in the first few years when the population size is small, or it will become persistent with a population size increasing, on average, by a factor of 1.5 each year.

## 3. A Branching Process with the Reproduction Law Following a Poisson Distribution

Both Fisher [3] and Haldane [4] used the Poisson distribution as the reproduction law to model reproductive success in their use of the branching process, i.e., the probability of an individual producing 0, 1, ..*k*, .. offspring is $f\left(k\right)=e^{-\lambda }\frac{\lambda^{k}}{k!}$. This is also used in epidemiological studies; i.e., an infected individual will pass the pathogen to 0, 1, 2, …k individuals with the probabilities following the Poisson distribution. One can estimate the parameter λ and then use the branching process to find the extinction probability and extinction time.

We know that the mean and variance of the Poisson distribution are both equal to λ. The mean and the variance can also be derived from the PGF below:

(28) $$G(s)=\sum_{k=0}^{Maxk}f\left(k\right)s^{k}=e^{-\lambda }+e^{-\lambda }\lambda s+e^{-\lambda }\frac{\lambda^{2}}{2!}s^{2}+e^{-\lambda }\frac{\lambda^{3}}{3!}s^{3}+\cdots$$

(29) $$G(s)=e^{-\lambda }\left(1+\lambda s+\frac{{\left(\lambda s\right)}^{2}}{2!}+\frac{{\left(\lambda s\right)}^{3}}{3!}+\cdots \right)=e^{-\lambda }e^{\lambda s}=e^{\lambda (s-1)}$$

(30) $$G^{\prime \left(s\right)}=e^{-\lambda }\lambda +2e^{-\lambda }\frac{\lambda^{2}}{2!}s+3e^{-\lambda }\frac{\lambda^{3}}{3!}s^{2}+\cdots +ne^{-\lambda }\frac{\lambda^{n}}{n!}s^{n-1}+\cdots =\lambda e^{\lambda (s-1)}$$

(31) $$G^{\prime \prime \left(s\right)}=2e^{-\lambda }\frac{\lambda^{2}}{2!}+6e^{-\lambda }\frac{\lambda^{3}}{3!}s^{1}+\cdots +n\left(n-1\right)e^{-\lambda }\frac{\lambda^{n}}{n!}s^{n-2}+\cdots =\lambda^{2}e^{\lambda (s-1)}$$

(32) $$E(X)=G\prime \left(1\right)=e^{-\lambda }\lambda \left(1+\lambda s+\frac{{\left(\lambda s\right)}^{2}}{2!}+\cdots \right)=\lambda$$

(33) $$G\prime \prime \left(1\right)=e^{-\lambda }\lambda^{2}\left(1+\lambda +\frac{\lambda^{2}}{2!}+\frac{\lambda^{3}}{3!}+\cdots \right)=e^{-\lambda }\lambda^{2}e^{\lambda }=\lambda^{2}$$

(34) $$Var(X)=G\prime \prime \left(1\right)+G\prime \left(1\right)-{\left[G\prime \left(1\right)\right]}^{2}=\lambda^{2}+\lambda -\lambda^{2}=\lambda$$

### 3.1. Extinction Probability Q

If λ≤1, then Q=1. If λ>1, then Q<1. We will first address the same question of extinction probability *Q*, and then deal with the extinction time *T*. From what we have learned, we need to solve Q=G(Q) and find the smallest non-negative root.

From Equation (29), we have

(35) $$Q=G\left(Q\right)=e^{\lambda \left(Q-1\right)}$$

In the critical case, when E(X)=λ=1, the only root for equation $Q=e^{\left(Q-1\right)}$ is Q=1; i.e., extinction is certain. This also implies that all cases with λ<1 should also have Q=1 because extinction would be even more likely with smaller λ. Thus, Q=1 for all λ≤1.

What would be the extinction probability under supercritical conditions with λ>1? Haldane [4] and Felsenstein [11] illustrated the solution with a λ only slightly larger than 1 (i.e., a small selection coefficient *s* = λ−1), so that one can expand the right-hand side of Equation (35) as a power series and drop high-order terms to obtain an approximate *Q*. This would not work with larger λ. There is no need for obtaining approximate solutions for *Q* when the reproduction law is Poisson, because *Q* has a closed-form solution expressed with a Lambert W function:

(36) $$Q=-\frac{1}{\lambda }W_{0}\left(-\lambda e^{-\lambda }\right)$$

where *W*_0_ is the principal branch of the Lambert W function [12]. $W_{0}(z)$ is defined as

(37) $$z=W_{0}\left(z\right)e^{W_{0}\left(z\right)}$$

*W*_0_ can be calculated in many numerical ways [12]; e.g., by using the ‘optim’ function in R or the Solver function in EXCEL. Table 2 lists *Q* values given different λ values. I have also included the extinction probability when the reproduction law follows a geometric distribution (Q_geometric_, the last column in Table 2). When λ≥1, *Q* has a simple closed-form expression of Q=1/R, where *R* is the mean for the geometric distribution equivalent to λ in a Poisson distribution; that is, R=(1−p)/p, where p is the only parameter in a geometric distribution. The Poisson distribution has its mean equal to its variance, but the geometric distribution has its variance greater than its mean and is therefore more appropriate for modeling differential reproductive success; i.e., many individuals produce 0 offspring, but a few produce many offspring. One could also use the negative binomial distribution to model the differential reproductive success, but *Q* does not have a closed-form expression with the negative binomial distribution. Both Poisson and geometric distributions are often used to model early colonization [13] or the early spread of infectious diseases [14,15,16].

Epidemiologists have documented the basic reproduction number (*R*_0_, which is equivalent to λ in Table 2) for various infectious diseases [14]. For COVID-19, *R*_0_ varies from 2 to 7 [17], which corresponds to a very small *Q*. Such a large *R*_0_ value implies that the infectious virus would spread quickly if the first ancestor did not experience bad luck and die instantly. For the Asian hornet *V. velutina*, empirical data are not sufficient to establish if the reproductive success follows the Poisson distribution. However, the mean number of offspring per individual, which is equivalent to λ, is 3.9 in France and 1.3 in South Korea [8]. Table 2 tells us that *Q* is about 0.58 in South Korea and 0.02 in France. As will be shown later, a λ value of 3.9 corresponds to a mean extinction time of 1.09. This means that the population will expand and spread rapidly unless the first colonizing queen experienced bad luck and failed to reproduce any daughter queens in her first generation in the new region.

The calculation above is also applicable to many evolutionary scenarios, such as the fate of a mutant in a haploid population or of a parthenogenetic individual arising from a sexual population. Imagine a large, stable, and genetically homogenous haploid population in which everyone is expected to produce one offspring to replace itself (λ=1). A new mutant arises with λ=1.01; i.e., it is fitter than other individuals in the population with a fitness differential s=0.01. Even such a beneficial mutation would have an extinction probability Q=0.98034 (Table 2). Its fixation probability is (1−Q)=1−0.98034≈0.02=2s. For s=0.001, Q=0.998002, 1−Q=0.002=2s. It is generally true that, for a small *s*, the fixation probability is 2*s* when the reproduction law follows a Poisson distribution.

The calculation is also applicable to the extinction probability and extinction time for a new parthenogenetic individual in a diploid sexual population. Such a parthenogenetic individual is supposed to have a two-fold advantage in fitness [18,19]; i.e., with λ=2 in contrast to an average sexual individual whose reproductive output is λ=1. The extinction probability for this new parthenogenetic individual is Q=0.20319 (Table 2).

One might be curious about why a λ greater than 1 (e.g., 1.1) could be associated with a large *Q*; e.g., 0.82391 for λ=1.1 (Table 2). In population genetics where λ is the absolute fitness of a mutant, the selection coefficient *s* = λ − 1 (against an implicitly assumed stable wile-type individual with a fitness of 1). When *s* is positive, then the mutant enjoys a selective advantage and should increase in number. However, that is a deterministic statement when the advantageous mutant is well represented. When population size is small, even a mutant with a high reproductive potential may perish without leaving any offspring [13]. Also note that *Q* is applicable only to one reproducing individual and its descendants. If we start with two reproducing individuals, then the probability that both will go extinct is Q^2^. Given λ=1.1 and 100 reproducing individuals, then the total extinction probability would be $0.82391^{100}=0.000000004$. Thus, with λ=1.1, if the founding individual is lucky enough to have its surviving descendants drifting up to 100, then the chance of total extinction is effectively 0. For the parthenogenetic individual with a two-fold fitness advantage, λ=2. If she could manage to leave 10 surviving offspring, then the chance of total extinction is $Q^{10}=0.20319^{10}=0.00000012$; i.e., effectively 0.

I wish to highlight the point that Q_geometric_ is substantially greater than *Q* (Table 2). This should have implications for the chance of success of individuals colonizing a new region and, by generalization, the chance of success of allopatric and parapatric speciation. In the extreme case of a bee colony with 1000 individuals but only one reproducing queen, the total extinction rate is *Q*, not Q^1000^.

### 3.2. Extinction Probability of a New Advantageous Allele in a Diploid Population

While our illustration above is applicable to an asexual haploid population, the conclusion is also true for diploid populations [4,11]. In a large population of wild-type individuals with AA genotypes, each individual produces 0, 1, 2, … k offspring with corresponding probabilities $p_{0},\;p_{1},\;p_{2},\;\ldots ,\;p_{k}$, respectively. What is *Q* for a new B allele that creates an AB heterozygote? If all alleles are neutral, then the probability of fixation of a new allele is simply its allele frequency; i.e., 1/(2*N*) for a new allele, where *N* is the population size of the diploid population. Thus, Q=1−1/(2N). What would be the extinction probability and extinction time when the B allele is advantageous?

An advantageous individual or allele can go extinct because of bad luck when the carriers of the advantageous allele are few. If the new beneficial allele B overcomes the bad luck and becomes frequent, then it tends to achieve fixation. This allows us to assume that, during the process of the allele going extinct, its frequencies are low, and it therefore exists in heterozygotes instead of BB homozygotes. For convenience, we may also assume that the B allele is dominant or codominant so that its beneficial effect will manifest in heterozygotes. Given these conditions, the extinction probability can be written in the form that we are already familiar with:

(38) $$Q=p_{0}+p_{1}Q+p_{2}Q^{2}+p_{3}Q^{3}+\cdots +p_{k}Q^{k}+\cdots$$

If we further assume that $p_{i}$ follows the Poisson distribution, then $Q=e^{\lambda (Q-1)}$ as we have derived before in Equation (35). The solution for *Q* and numeric estimates for *T* and *Var*(*T*) have already been derived and illustrated in the previous section. The assumption of independent propagation of the new allele B in the population is reflected in those terms with $Q^{2},\;Q^{3},\;Q^{4},\ldots$ in Equation (38). In other words, all B alleles exist in AB heterozygotes and independently and therefore have the same extinction probability as the first AB heterozygote created by the mutation. With Equation (36), we can readily compute *Q*, *T*, and *Var*(*T*) for any λ. The extinction probability for the B allele has already been calculated in Table 2 with different selective advantages against an implicit wild-type population with λ=1. The λ values in Table 2 minus 1 are the selection coefficients.

I have illustrated the derivation and calculation of the extinction probability of a new advantageous allele B in a haploid population. This has also been performed in a large diploid population by assuming that, because of the large population, allele B would almost always exist in heterozygotes, so different B alleles still reproduce independently and follow the same reproduction law. If it becomes frequent enough to form a non-negligible number of BB individuals in a large population, it must have overcome bad luck and will persist. In other words, we can ignore BB individuals in deriving the extinction probability in a large population.

With a small population, BB individuals will quickly emerge. What is the probability of extinction of the new advantageous allele B in a finite population when BB individuals do appear in non-negligible numbers? A convenient specification of fitness for the three genotypes is $w_{BB}=1,\;w_{AB}=1-hs,\;w_{AA}=1-s$. Unfortunately, such a specification violates a fundamental assumption of the branching process; i.e., the B allele should be propagated identically and independently without being affected by which allele it is associated with in a diploid. For this reason, Fisher [3,20] avoided dominance, as did Haldane [4] and Wright [21], although the latter two estimated the survival probability of a fully recessive allele, which, in heterozygotes, is essentially neutral. Moran [22] used the branching process to derive the survival probability of a mutant in a finite haploid population where the problem of dominance does not arise. In order to accommodate selection and dominance, Kimura [23,24] used diffusion models to accommodate dominance and selection. He did not “fix” the classical branching process. Instead, he abandoned the approach of tracking individual lineages and focused on tracking allele frequencies as a stochastic process. We need to go beyond the classic branching process to handle such scenarios. To maintain continuity, I postpone this issue to Section 5 of the paper.

### 3.3. Extinction Time

Let us first consider the subcritical condition with λ=0.7. This way, we can compare the results to those in the first example where E(X)=0.7, Var(X)=0.61. For a Poisson distribution, E(X)=Var(X)=λ=0.7. Thus, we expect the mean and variance of the extinction time to be like those in our first example. Again, here are the first few $q_{t}$ values.

(39) $$q_{1}=G(q_{0})=G(0)=e^{-\lambda }\approx 0.4966$$

(40) $$q_{2}=G(q_{1})=G\left(e^{-\lambda }\right)\approx 0.7030$$

(41) $$q_{3}=G(q_{2})\approx 0.8123$$

(42) $$q_{4}=G(q_{3})\approx 0.8769$$

In all subcritical cases, $q_{t}$ will increase monotonously towards 1. The probability that extinction happens exactly at time *t*—i.e., $\mathrm{Pr}\left(T=t\right)$—can also be calculated as before:

(43) $$\mathrm{Pr}(T=1)=q_{1}-q_{0}=0.4966$$

(44) $$\mathrm{Pr}(T=2)=q_{2}-q_{1}=0.2064$$

(45) $$\mathrm{Pr}(T=3)=q_{3}-q_{2}=0.808-0.7=0.1093$$

(46) $$\mathrm{Pr}(T=4)=q_{4}-q_{3}=0.876-0.808=0.0646$$

Given the distribution, we can calculate *T* with the two formulae that we have used before for subcritical conditions:

(47) $$T=\sum_{t=1}^{\infty }t\mathrm{Pr}(T=t)=1\times 0.4966+2\times 0.2064+\cdots \approx 2.3762$$

(48) $$T=\sum_{t=0}^{\infty }\left(1-q_{t}\right)=\left(1-0\right)+\left(1-0.4966\right)+\left(1-0.7030\right)+\cdots \approx 2.3762$$

What would be the extinction time *T* and its *Var*(*T*) under supercritical conditions? *T* can be calculated the same way by using Equation (21). *Var*(*T*) can also be calculated with Equation (27). For example, if λ=1.1, then T=4.102, Var(T)=33.550. Table 3 lists the *q_t_* and (*q_t_* − *q*_*t*−1_) values needed to compute *T* and *Var*(*T*) for λ=1.1. We already have listed Q=0.82391 for λ=1.1 in Table 2, which is consistent with the *q_t_* column in Table 3 that comes very close to this number when *t* reaches 1000 generations.

In our numeric illustration of the supercritical condition with λ=1.1, *T* is longer than the subcritical condition with λ=0.7. However, it is possible for a smaller *T* under supercritical conditions; for example, if λ=2, then T=1.545, Var(T)=0.953. This is because the extinction time is conditional on extinction. An individual that reproduces with λ=2 will either have bad luck and go extinct quickly (i.e., a small *T*), or increase in population size rapidly and never go extinct.

The invasive Asian hornet, *V. velutina*, has a mean number of 3.9165 offspring per individual in France [8]. If we assume that the reproductive success follows the Poisson distribution, then T=1.089, Var(T)=0.097. Thus, the invasive individuals should either have bad luck and go extinct in the first generation with the extinction probability Q=0.02 (Table 2) or will spread quickly and never go extinct. Since its estimated landing around 2002 [8], the invasive hornet has spread beyond France into many European countries.

## 4. Come Back to the Galton Questions

Galton asked two questions, given the following conditions. *N* adult men each have a unique surname. In each generation, each man produces *k* adult sons with probability *a_k_*. Galton limited *k* to a maximum of five. His two questions are as follows. First, what is the chance of a surname whose bearers will perish after *t* generations? Second, after *t* generations, how many surnames will have *m* (=0, 1, 2, …,) bearers?

The PGF for addressing Galton’s two questions is similar to our first example:

(49) $$\begin{array}{c} G(s)=a_{0}+a_{1}s+a_{2}s^{2}+a_{3}s^{3}+a_{4}s^{4}+a_{5}s^{5}\\ G^{\prime }(s)=a_{1}+2a_{2}s+3a_{3}s^{2}+4a_{4}s^{3}+5a_{5}s^{4}\\ G^{\prime \prime }(s)=2a_{2}+6a_{3}s+12a_{4}s^{2}+20a_{5}s^{4}\\ E(X)=G^{\prime }\left(1\right)=a_{1}+2a_{2}+3a_{3}+4a_{4}+5a_{5}\\ Var(X)=G^{\prime \prime }\left(1\right)+G^{\prime }\left(1\right)-{\left[G^{\prime }\left(1\right)\right]}^{2} \end{array}$$

Suppose we have $a_{0}=0.1,a_{1}=0.3,a_{2}=0.25,a_{3}=0.15,a_{4}=0.1,a_{5}=0.1$. The mean number of sons produced by a man, *E*(*X*), and the associated variance, *Var*(*X*), are

(50) $$\begin{array}{c} {E(X)=G}^{\prime }\left(1\right)=2.15\\ Var(X)=G^{\prime \prime }\left(1\right)+G^{\prime }\left(1\right)-{\left[G^{\prime }\left(1\right)\right]}^{2}=2.1275 \end{array}$$

This is a supercritical condition, so the population size will more than double itself each generation in a deterministic model. The extinction probability Q<1. Solving the equation $Q=G\left(Q\right)$ gives us Q=0.1519416038. The probability of extinction by generation *t* is $q_{t}$ which we have calculated recursively in previous examples. In mathematics, this $q_{t}$ is calculated as the t-fold composition of *G* or the tth iterate of G:

(51) $$q_{t}=G^{\left(t\right)}(0)$$

This function does not have a closed form, which is why we resorted to computing $q_{t}$ recursively in previous examples. For the first few generations:

(52) $$\begin{array}{c} q_{0}=0\\ q_{1}=G\left(q_{0}\right)=G\left(0\right)=a_{0}=0.1\\ q_{2}=G\left(q_{1}\right)=G\left(0.1\right)=0.132661\\ q_{3}=G\left(q_{2}\right)=0.1445833203 \end{array}$$

Thus, after one generation, 10% of the *N* surnames are lost. At generation 23, the proportion of surnames lost has already reached *Q* (=0.1519416038). After that, all remaining surnames would already have escaped bad luck and would persist. The extinction time *T* is only 1.547654, with *Var*(*T*) = 0.916671. This means that ~15.2% of lost surnames must have been lost in early generations, otherwise a few generations of rapid increase in population size (more than doubling in each generation) would secure their permanent existence.

Thus, to address Galton’s first question, as long as $a_{i}$ values and *t* are given, it is easy to recursively compute $q_{t}$, which is the proportion of surnames that have gone extinct by generation *t* (Table 4). For example, about 14.9% of the surnames are expected to perish by generation 4 under the extremely favorable condition with μ=2.15.

Galton’s second question is trickier. At generation *t*, how many of those starting *N* surnames are carried by 0, 1, 2, …, men? A similar question has been addressed with a different approach [25]. Here, I illustrate the answer using the classic branching process.

**Figure 2:**
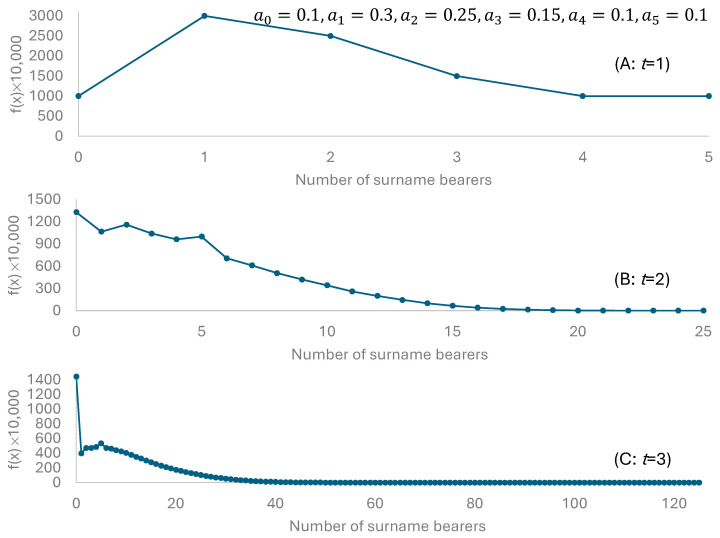
Probability distribution of surnames with 0, 1, 2, … bearers after the first three generations, multiplied by 10,000. Reproduction is specified by *a_k_* values, with *μ* = 2.15.

Our illustrative example has $a_{0}=0.1,\;a_{1}=0.3,\;a_{2}=0.25,\;a_{3}=0.15,\;a_{4}=0.1,\;a_{5}=0.1$. Suppose we have *N* = 10,000 men each bearing a unique surname at time *t =* 0. At time *t* = 1, we expect 1000 ($=N\cdot a_{0}$) surnames to perish. We expect 3000, 2500, 1500, 1000 and 1000 surnames, respectively, to have 1, 2, 3, 4 and 5 bearers, respectively (Figure 2A). At time *t* = 2, the proportion of perished surnames is expected to increase from 10% to 13.277% (Figure 2B). The distribution is also wider (Figure 2B), because there are some lucky surnames that might have as many as 25 (=5×5) bearers after the second generation. For example, the man with a surname Clark might produce 5 sons in the first generation, and these sons each produce 5 sons in the second generation. Now the surname Clark has 25 bearers. However, the probability of having such good luck is small; that is, the product of $a_{5}$ from the first generation multiplied by $a_{5}^{5}$ in the second generation (i.e., each of the five sons independently produces five sons in the second generation). There are about 1159 surnames with two bearers after the second generation (Figure 2B). After the third generation, the largest possible number of bearers of a surname is 125 (=5^3^) (Figure 2C). However, the probability for such an event to occur is 10^−31^. The total probability of having a surname with 50 or more bearers is only 3.24^−4^. Note that this is under extremely favorable conditions with μ=2.15. In a stable population with μ=1 (e.g., when $a_{0}=0.45,\;a_{1}=0.35,\;a_{2}=0.15,\;a_{3}=0.06,\;a_{4}=0.03,\;a_{5}=0.01$), then the surnames lost would be 45% after the first generation, 64% after the second generation, and 78% after the third generation. The total probability for surnames with 10 or more bearers is only 2.97^−3^ after three generations with this new set of $a_{k}$ values.

## 5. Extinction Probability and Extinction Time for a New Allele in a Small Population

In a previous section, I have briefly dealt with a diploid population of AA homozygotes in which an advantageous mutation creates an AB heterozygote, and asked the question of the extinction probability of this B allele. I assumed a population so large that the B allele would exist only in heterozygotes. In a small population, BB homozygotes will arise. This last part will treat a process that is analogous to a branching process but violates the fundamental assumption of the independence (or non-interference) of the branching process.

I will start with an extreme case. The population has only one AA individual that produces exactly one offspring per generation by selfing. An advantageous mutation changed this AA individual to AB. What is the extinction probability of the advantageous B allele? Because the population size is fixed at 1, selection cannot operate in the diploid phase, so the selective advantage must be materialized in the haploid phase.

We might think that, in this population of a single selfing heterozygote of AB genotype that produces exactly one offspring per generation, the probabilities for 0, 1 or 2 copies of the new B alleles (i.e., *N_B_* = 0, 1 or 2) in the next generation are 0.25, 0.5, and 0.25, respectively, assuming random association of gametes and no selection. We may unwittingly write down something foolish as follows:

(53) $$\begin{array}{c} f\left(N_{B}=0\right)=0.25;\\ f\left(N_{B}=1\right)=0.5;\\ f\left(N_{B}=2\right)=0.25\\ G(s)=0.25+0.5s+0.25s^{2}\\ Q=G\left(Q\right)=0.25+0.5Q+0.25Q^{2} \end{array}$$

We instantly know that the formulation of *Q* above is wrong. If the offspring in the next generation is BB, then the B allele is fixed and will never go extinct. The last term, 0.25*Q*^2^, assumes that the two B alleles propagate independently and both have the probability of *Q* of independent extinction in a BB zygote, which is absurd. The reason for this is that the branching process, as explicitly defined [5,6], involves reproducing objects that produce offspring independently of each other. The objects could be individuals or alleles, but they should not interfere with each other. It should not be used where the extinction probability of an individual or an allele depends on other individuals or alleles. When two B alleles come together to form a BB offspring in a population of a single individual, the extinction probability becomes 0. Thus, our problem violates the most fundamental assumption of the branching process.

On the other hand, our single-individual population that produces exactly one individual per generation by selfing does have some similarity to the branching processes we have illustrated before. For the new selfing AB heterozygote, it has a probability of 0.25 of having an AA offspring (when allele B is neutral), which implies extinction of the B allele. It has a probability of 0.5 of having an AB offspring that has the same extinction probability *Q* as the parent. It has a probability of 0.25 of having a BB offspring, which implies fixation (i.e., the extinction probability is 0). Therefore

(54) Q=0.25·1+0.5·Q+0.25·0=0.25+0.5·Q

The solution is Q=0.5, which is intuitively obvious. This example illustrates an important point. We do not always need to express *Q* in the form of a PGF in order to obtain *Q*.

If the new B allele is fitter than the A allele, then the probability of having an AA, AB, or BB offspring for the next generation will not be 0.25, 0.5, and 0.25, respectively. This would lead to a more general expression of *Q* and its solution:

(55) $$\begin{array}{c} Q=P_{AA}\cdot 1+P_{AB}\cdot Q+P_{BB}\cdot 0=P_{AA}+P_{AB}Q\\ Q=\frac{P_{AA}}{1-P_{AB}} \end{array}$$

Because the population has only one individual in each generation, any selection would have to occur in the haploid gamete stage. Suppose the fitness is 1 for the original A allele and 1 + *s* for the mutant B allele during the haploid phase. The change in the allele frequency of A (designated by *p*) is a special case of a more general formula for multiple alleles at a single locus [26]:

(56) $$\Delta p=-\frac{sp\left(1-p\right)}{1+s\left(1-p\right)}$$

If *s* = 0.01, then the expected allele frequency for allele A in the gamete pool after the gamete selection would be 0.497512 instead of 0.5. The AA genotype frequency would now be 0.247519 instead of 0.25. The extinction probability *Q* for the new and beneficial allele B is now reduced from 0.5 to 0.495025.

For a population with 1 to 16 individuals following the Wright-Fisher model [20,27] but without selection, it is convenient to calculate *Q*, *T* and *Var*(*T*) by using Markov chains. For larger populations, the diffusion approximation derived by Kimura and Ohta [28,29] is quite accurate. Table 5 presents a comparison between the exact calculation by the Markov chain and the approximation by using equations in Kimura and Ohta [28,29]. The difference is already small with *N* = 16 and will become smaller with larger *N.*

## 6. Recent Extensions and Applications

The largest number of illustrations and potential biological applications of the branching process is available in a book [30]. Recent studies have extended the classical branching process in several ways. The first is parameter estimation. Equation (1) lists three parameter values, 0.5, 0.3, and 0.2 that define the reproduction law needed for the branching process. The λ parameter in the Poisson distribution is also a parameter that can be estimated from empirical data. Such estimation can be carried out either nonparametrically or by maximum likelihood or Bayesian approaches [31,32], the last being particularly relevant for small populations with limited empirical data.

Suppose a parthenogenetic female with discrete generations was introduced on a large island and then forgotten. The population size has grown so rapidly as to attract sufficient attention to have the population size (*Z*) monitored, starting at time t. In four consecutive generations, $Z_{t+0}=138057,\;Z_{t+1}=248503,\;Z_{t+2}=546707,\;Z_{t+3}=1158576$. Assuming that the reproduction law follows a Poisson distribution, the maximum likelihood estimate of λ [32] is

(57) $$\lambda =\frac{\sum_{i=1}^{3}Z_{t+i}}{\sum_{i=0}^{2}Z_{t+i}}=\frac{248503+546707+1158576}{138057+248503+546707}=2.09349093$$

Given the reproduction law following a Poisson distribution, the extinction probability *Q* is fully determined by λ. Using the method illustrated in Table 2, we obtain Q=0.179463. The maximum likelihood estimator of the number of generations from the first release of the parthenogenetic female [33] is

(58) $$N=\frac{ln\left(\left(1-Q\right)Z_{t+3}+Q\right)}{\ln\lambda }=18.63$$

Thus, the first parthenogenetic female was released about 19 generations ago. Such parameter estimation helps us extract ecologically relevant information from data. For discrete generations, Monte Carlo methods have been used to validate the parameter estimators [32]. For continuous-time branching processes, one could potentially use the Gillespie algorithm [34] for simulation purposes. This involves probability theory more advanced than what is covered in this paper.

Second, the branching process itself can be generalized. In our illustration, the same reproduction law is applied to all individuals. However, parental reproductive success could be correlated with offspring reproductive success. Such a scenario implies that some lineages are less likely to go extinct than others. For example, successful mothers tend to have successful daughters and so on. One can modify the reproduction law to accommodate this parent-offspring correlation in reproductive success [35]. Also, my illustrative examples are for populations with discrete generations. Under certain circumstances, the branching process can also accommodate populations with overlapping generations [35].

The theory of multi-type branching processes was developed a long time ago [5,6], but has only recently been applied to solve practical biological problems on the consequences of dormancy on population demography. A haploid population was implicitly assumed. The empirical challenge is to determine what environmental factors trigger the switch between the active and dormant state and whether the probability of switching between the two states depends quantitatively on the degree of environmental harshness.

The branching process has not been used for analyzing demographic trends, which has led to misconceptions such as the claim of a decreasing White population size. According to the census data of the US government, available at https://www.census.gov/data/tables/2024/demo/fertility/women-fertility.html (accessed on 6 December 2025), the percentage of White women aged 45–50 with 0, 1, 2, 3, 4, and 5+ children is 16.2, 16, 36.1, 20.2, 7.8, and 3.8, respectively. Fitting a Poisson distribution to yields a λ=2.0043. Thus, the White population in the US will continue to grow, albeit slower than Black and Hispanic populations, which have λ=2.1967 and 2.2899, respectively. In contrast, λ=1.7648 for the Asian population, which means that the Asian population would perish rapidly without immigration. The branching process, when applied to small local ethnic populations, would provide good predictions on the extinction of such small populations and on ethnic compositions in the future to serve as the basis for government policies on ethnic diversity.

## 7. Conclusions

This paper aims to popularize the branching process with the objective of highlighting its potential application. The illustrative examples can be easily extended to more complicated scenarios. For example, the reproduction law was illustrated with the empirically determined distribution and with the Poisson distribution, but one can replace the reproduction law with a geometric distribution or a negative binomial distribution to model more unequal reproductive success among individuals; e.g., most individuals fail to reproduce, but a few produce many offspring.

## Figures and Tables

**Table 1:** Numerical method for computing extinction time *T* and its variance.

T	*q*_t_	*q*_t_ − *q*_t − 1_	1 − *q*_t_	(2*t* + 1)(1 − *q*_t_)
0	0		1	1
1	0.5	0.5	0.5	1.5
2	0.7	0.2	0.3	1.5
3	0.808	0.108	0.192	1.344
4	0.872973	0.064973	0.127027	1.143245
5	0.914308	0.041335	0.085692	0.94261
6	0.941484	0.027176	0.058516	0.760704
…	…	…	…	…
37	0.999999	3.75 × 10^−7^	8.74 × 10^−7^	6.56 × 10^−5^
38	0.999999	2.62 × 10^−7^	6.12 × 10^−7^	4.71 × 10^−5^
39	1	1.84 × 10^−7^	4.28 × 10^−7^	3.38 × 10^−5^
40	1	1.28 × 10^−7^	3 × 10^−7^	2.43 × 10^−5^
…	…	…	…	…
92	1	1.22 × 10^−15^	2.55 × 10^−15^	4.72 × 10^−13^
93	1	0	1.89 × 10^−15^	3.53 × 10^−13^
94	1	0	0	0

**Table 2:** Relationship between λ and *Q* for *λ* > 1 when the reproduction law follows a Poisson distribution. The calculation of W_0_ is explained in the text. The last column is the extinction probabilities when the reproduction law follows a geometric instead of a Poisson distribution, with the same mean as λ.

λ	$z=-\lambda e^{-\lambda }$	W_0_(z)	Q	Q_geometric_
1	−0.36788	−1	1	1
1.01	−0.36786	−0.99014	0.98034	0.99900
1.1	−0.36616	−0.90630	0.82391	0.99010
1.2	−0.36143	−0.82353	0.68627	0.83333
1.3	−0.35429	−0.75013	0.57702	0.76923
1.4	−0.34524	−0.68461	0.48901	0.71429
1.5	−0.33470	−0.62581	0.41720	0.66667
2	−0.27067	−0.40637	0.20319	0.5
3	−0.14936	−0.17856	0.05952	0.33333
4	−0.07326	−0.07931	0.01983	0.25
5	−0.03369	−0.03489	0.00698	0.2
10	−0.00045	−0.00045	0.00004	0.1

**Table 3:** The *q_t_* and *q_t_* − *q*_*t*−1_ for computing extinction time *T* and its variance *Var*(*T*) for *λ* = 1.1.

T	*q_t_*	*q_t_* − *q*_*t*−1_
0	0.0000000000	
1	0.3328710837	0.332871084
2	0.4800611401	0.147190056
3	0.5644334773	0.084372337
4	0.6193261945	0.054892717
5	0.6578744403	0.038548246
6	0.6863702213	0.028495781
7	0.7082254834	0.021855262
8	0.7254580952	0.017232612
…	…	…
999	0.8238658564	0
1000	0.8238658564	0

**Table 4:** *q_t_* values for *a*_0_ = 0.1, *a*_1_ = 0.3, *a*_2_ = 0.25, *a*_3_ = 0.15, *a*_4_ = 0.1, *a*_5_ = 0.1.

T	$q_{t}$	$q_{t}-q_{t-1}$
0	0	
1	0.1000000000	0.1000000000
2	0.1326610000	0.0326610000
3	0.1445833203	0.0119223203
4	0.1491044603	0.0045211400
5	0.1508434063	0.0017389461
…	…	…
23	0.1519416037	0.0000000001
24	0.1519416038	0.0000000000
25	0.1519416038	0.0000000000

**Table 5:** Comparison of accuracy between the exact estimate of extinction and fixation time (${\bar{t}}_{Loss}$ and ${\bar{t}}_{Fix}$, respectively, columns 3 and 4) by using the Markov chain and the approximate estimate using equations from Kimura and Ohta [28,29] (columns 5 and 6). *N* is the population size and *p* is the frequency of the new B allele in the population.

N	*p*	${\bar{t}}_{Loss}$	${\bar{t}}_{Fix}$	${\bar{t}}_{Loss}$	${\bar{t}}_{Fix}$
1	1/2	2	2	2.7726	2.7726
2	1/4	2.9931	5.7793	3.6968	6.9044
4	1/8	4.0768	13.5534	4.7530	14.9555
16	1/32	6.5006	61.0634	7.1551	62.9894

## Data Availability

Not applicable
